# Mortality Among Hospitalized Patients With Pleural Effusions. A Multicenter, Observational, Prospective Study

**DOI:** 10.3389/fmed.2022.828783

**Published:** 2022-02-24

**Authors:** Eleftherios Markatis, Garifallia Perlepe, Andreas Afthinos, Konstantinos Pagkratis, Charalampos Varsamas, Eleftheria Chaini, Ilias C. Papanikolaou, Konstantinos I. Gourgoulianis

**Affiliations:** ^1^Pulmonary Department, Corfu General Hospital, Corfu, Greece; ^2^Department of Respiratory Medicine, Faculty of Medicine, University of Thessaly, Larissa, Greece

**Keywords:** mortality, prognostic factors, pleural effusion, hospitalized patients (inpatients), survival

## Abstract

**Background:**

Data regarding the prognostic significance of pleural effusion (PE) are scarce.

**Objective:**

Explore the impact of PE on mortality among hospitalized patients.

**Methods:**

Multicenter prospective observational study. Patients that underwent computed tomography (thorax and/or abdomen) and in which PE was detected, were admitted to the study. PE was classified by size on CT, anatomical distribution, diagnosis, and Light's criteria. Charlson comorbidity index (CCI), APACHE II, and SOFA score were calculated. Mortality at 1 month and 1 year were recorded.

**Results:**

Five hundred and eight subjects, mean age 78 years. Overall mortality was 22.6% at 1 month and 49.4% at 1 year. Bilateral effusions were associated with higher mortality than unilateral effusions at 1 month (32 vs. 13.3%, *p* = 0.005) and large effusions with higher mortality than small effusions at 1 year (66.6 vs. 43.3%, *p* < 0.01). On multivariate analysis age, CCI, APACHE II, SOFA score, and bilateral distribution were associated with short-term mortality, while long-term significant predictors were CCI, APACHE II, SOFA, and malignant etiology. Exudates (excluding MPE) exhibited a survival benefit at both 1 month and 1 year but due to the smaller sample, fluid characteristics were not included in the multivariate analysis.

**Conclusions:**

Pleural effusion is a marker of advanced disease. Mortality is higher within the first month in patients with PEs related to organ failure, while patients with MPE have the worst long-term outcome. Independent predictors of mortality, apart from CCI, APACHE II, and SOFA scores, are age and bilateral distribution in the short-term, and malignancy in the long-term.

## Background

Pleural effusion (PE) is a common clinical condition, arising from a variety of systemic, malignant, infectious, and inflammatory diseases. It affects 1.5 million patients per year in the USA (1) and 200,000–250,000 in the UK (2) with an increasing burden following an aging population with more comorbidities (3). It has been established that PE significantly affects prognosis and mortality, depending on etiology. This applies to patients with malignant pleural effusion (MPE) whose mean survival is 1.5–9 months, to patients with pleural sepsis, and patients with acute decompensated heart failure (HF) (4–8). However, the impact of PE on outcomes and specific mortality of hospitalized patients has not been adequately addressed. In particular, Kookoolis and colleagues, in a small retrospective study of 104 patients in whom pleural effusion was found on plain chest radiography during admission, report mortality of 15% at 30 days and 32% in 1 year in those patients. Risk factors among those patients, of which only 1 out of 10 underwent a diagnostic puncture, were age, the severity of disease based on Apache score, malignancy, and underlying lung disease (9).

Debiasi and colleagues from the same center studied prospectively 308 patients hospitalized in internal medicine wards. All patients underwent diagnostic puncture and mortality was higher in patients with malignant effusion (37% at 30 days, 77% at 1 year). However, high mortality was also highlighted in patients with bilateral effusions regardless of etiology compared to unilateral effusions (mortality 47% at 30 days, 69% at 1 year) (10).

Finally, Walker and colleagues prospectively studied 356 patients with non-malignant pleural effusions in a single center. All patients underwent thoracentesis. Cardiac, renal, and hepatic failure were associated with significant 1-year mortality (50, 46, and 25%, respectively), while bilateral and transudative effusions were associated with worse prognosis (57 and 43% 1-year mortality rate, respectively) (11). The purpose of the present study is to investigate the short-term and long-term effect of PE on mortality, and possible correlations with the size, the location, and the etiology of the effusion as well as clinical severity scores.

## Methods

We conducted a prospective multicenter observational study in Corfu General Hospital Pulmonary Department and University of Larissa Pulmonary Department. Successive patients hospitalized between January 2018 and January 2020 that underwent computed tomography of the thorax and/or abdomen and in which PE was detected, were admitted to the study, regardless of etiology. The study protocol was approved by the respective ethics committees and study participants gave written informed consent.

Upon recruitment in the study, for each subject, we recorded demographics, smoking habit, Charlson comorbidity index (CCI), department in which subjects were admitted, main diagnosis of admission (ICD-10), and severity of disease (calculated by APACHE II and SOFA scores).

Further, PEs were quantified by size based on the division of the hemithorax on computed tomography (CT) into 4 quadrants as small (0–25%), moderate (25–50%), and large (50–100%) by the mid-clavicular line. In cases of doubt, the small effusion was up to 3 cm in size and the medium >3 cm up to 10 cm. In cases where the atelectatic lung was surrounded by fluid, this was counted in the total size of the effusion. This method has been described by other researchers to increase agreement on the classification of collections by size among clinicians (12). We also recorded whether effusions were unilateral or bilateral. In the latter case, the size of the largest collection was calculated. Type of CT (thorax, contrast-enhanced, computed tomography pulmonary angiography, and abdominal) was recorded in all cases. The effusions were not necessarily tapped for inclusion in the study. A diagnostic puncture was performed if deemed necessary. In these cases, diagnosis and treatment depended on the best medical practices and the judgment of the treating physician. If a diagnostic puncture was performed, Light's criteria were applied. The definite etiology of the effusion was determined by two pulmonary physicians. Electronic medical records of the respective hospitals were used to retrieve data on the survival of the patients in 1 month and 1 year, as well as the total days of hospitalization and other adverse outcomes.

### Statistical Analysis

For continuous variables, the mean, standard deviation, and range or median, 25th and 75th percentiles, and range were used after testing for normal distribution. The continuous variables were tested for normality using the Shapiro-Wilk test. For categorical variables, the frequencies and percentages are presented. SOFA score and APACHE II score were analyzed as categorical variables (SOFA: 0–1, 1–2, 2–3, 3–4, 4–5, >5 and APACHE: 0–4, 5–9, 10–14, 15–19, 20–24, 25–29). Univariate logistic regression was performed to identify statistically significant variables associated with 1-month and 1-year mortality. Then, all the statistically significant variables except the “transudate vs. exudate” variable were used for the construction of a model using multivariate logistic regression. For the construction of the model backward, stepwise selection approaches were used. The variable “transudate vs. exudate” was excluded due to the small number of observations compared to the other variables (201/508). Kaplan-Meier curves are presented regarding 1-month and 1-year survival. A *p* < 0.05 was considered statistically significant. All statistical analyses were performed using Stata/ IC version 15.1.

## Results

A total of 508 subjects were included in the analysis. Table 1 presents the demographics and the characteristics of pleural effusions. The mean age of the patients in our study was 78 years and the majority of patients were admitted to Pulmonary Departments with median hospitalization ranging from 8 to 12.5 days, while most patients underwent a thoracic CT. Pleural effusions were mostly small-sized, equally unilateral, or bilateral. When thoracentesis was performed exudates were more common (*n* = 160, 79.65%). Heart failure, malignant pleural effusion, and pleural infection were the leading diagnosis. Organ failure (liver, renal) and other exudates followed. Descriptive statistics of study subjects separated by outcome and short/ long term prognosis are shown in Table 2.

**Table 1:** Demographics and characteristics of pleural effusions (*n* = 508).

Age (years)	78, range 67–85
Male	292 (57.48%)
Smoking	298 (58.66%)
Charlson comorbidity index	5, range 3–5.5
Apache score	10, range 7–15
Sofa score	2, range 1–3
**Department of admission**	
Pulmonary department	312 (61.42%)
Internal medicine	108 (21.26%)
Surgical department	40 (7.87%)
Cardiology department	36 (7.09%)
Intensive care unit	12 (2.36%)
**Days of admission**	
Heart failure	10
Malignant pleural effusion	8
Pleural infection	10
Organ failure	10.5
Pulmonaty embolism	12
Connective tissue diseases	8.5
Tuberculosis	12.5
Other exudates	10
Multiple benign etiologies	13
**Type of CT**	
Thorax	278 (54.7%)
Abdominal	92 (18.1%)
CTPA^∧^	78 (15.35%)
Thorax & abdominal	60 (11.81%)
**Distribution**	
Unilateral	255 (50.2%)
Bilateral	253 (49.8%)
**Size of effusion**	
Small	277 (54.53%)
Medium	138 (27.17%)
Large	93 (18.31%)
Thoracentesis	201 (39.57%)
Transudate	41 (20.4%)
Exudate	160 (79.6%)
**Diagnosis**	
Heart failure	158 (31.1%)
Malignant pleural effusion	112 (22.05%)
Pleural infection	90 (17.72%)
Organ failure	44 (8.66%)
Other exudates^*^	37 (7.28%)
Pulmonary embolism	24 (4.72%)
Multiple benign etiologies	23 (4.53%)
Connective tissue diseases	16 (3.15%)
Tuberculosis	4 (0.79%)

∧ *CTPA, computed tomography pulmonary angiography,*

* *post coronary artery bypass graft, post-surgery, pancreatic disease, abdominal abscess, hemothorax, drug related, undiagnosed*.

**Table 2:** Comparative characteristics of subjects based on short and long-term outcome.

	**1 month outcome**		**1 year outcome**	
	**Survivors**	**Non-survivors**	**Survivors**	**Non-survivors**
Age years	75 (65–84)	83 (72–88)	73 (60–82)	79 (71–85)
In hospital days	10 (6–15)	10.5 (7–17.25)	10 (6–15)	10 (6–15)
CCI	4.0 (3–5)	5.0 (5–6)	4 (2–5)	5 (4–6)
APACHE II score	10.0 (5–13)	15.0 (13.75–19)	8 (4–12)	10 (7–15)
SOFA score	1.0 (1–3)	3.0 (3–4.25)	1 (1–2)	2 (1–3)
Male sex	223 (57)	68 (60)	143 (56)	80 (59)
Smoking	224 (57)	73 (64)	146 (57)	78 (57)
**Size**				
Small	218 (55)	57 (50)	158 (62)	62 (46)
Moderate	109 (28)	29 (25.5)	68 (26)	40 (29)
Large	66 (17)	28 (24.5)	31 (12)	34 (25)
**CT**				
Thorax wo contrast	142 (36)	53 (46)	91 (36)	51 (38)
Thorax with contrast	115 (29)	27 (24)	77 (30)	38 (28)
CTPA	71 (18)	7 (6)	50 (19)	21 (15)
Abdomen	65 (17)	27 (24)	39 (15)	26 (19)
Unilateral /bilateral	215 (55)	33 (29)	140 (54)	82 (60)
	178 (45)	81 (71)	117 (46)	54 (40)
Loculation	131 (33)	32 (28)	82 (32)	49 (36)
Thoracentesis	168 (43)	33 (29)	101 (39)	67 (49)
Exudate/transudate	138 (82)	21 (64)	85 (84)	54 (80)
	30 (18)	12 (36)	16 (16)	13 (20)

*Continuous variables are depicted as median with interquartile range (25–75) in parenthesis and categorical outcomes as absolute n with% frequency in parenthesis. Charlson comorbidity index, APACHE II and SOFA scores are presented in this table as continuous variables. CCI, Charlson comorbidity index; CT, computed tomography; CTPA, computed tomography pulmonary angiography; PPE, parapneumoic effusion; CTD, connective tissue disease; APACHE II, acute physiology and chronic health evaluation; SOFA, sequential organ failure assessment*.

**Figure 1:**
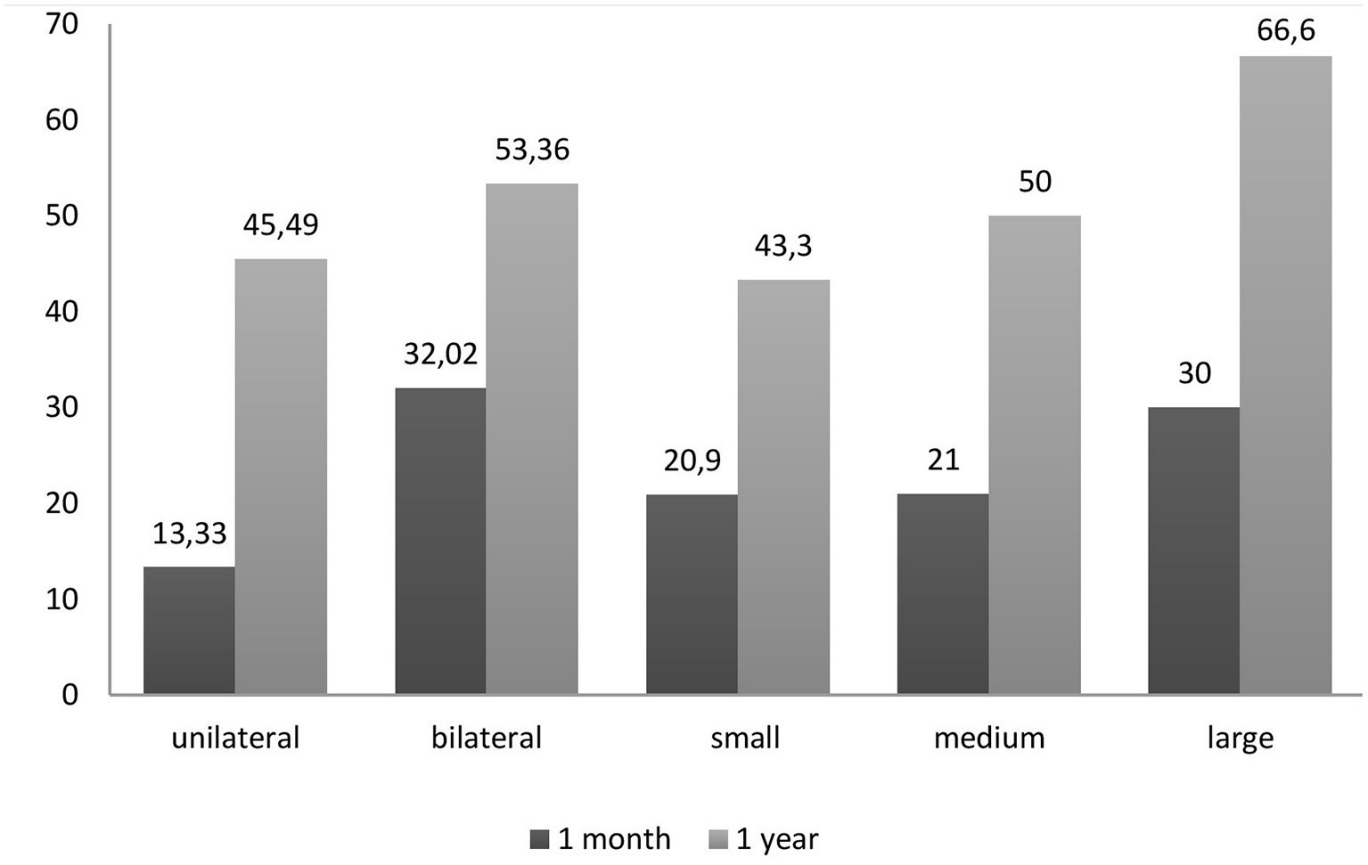
Percent mortality based on the distribution and size of the PE. Patients with large effusions exhibited higher mortality than patients with small effusions, while patients with bilateral effusions exhibited higher mortality than patients with unilateral effusions.

**Figure 2:**
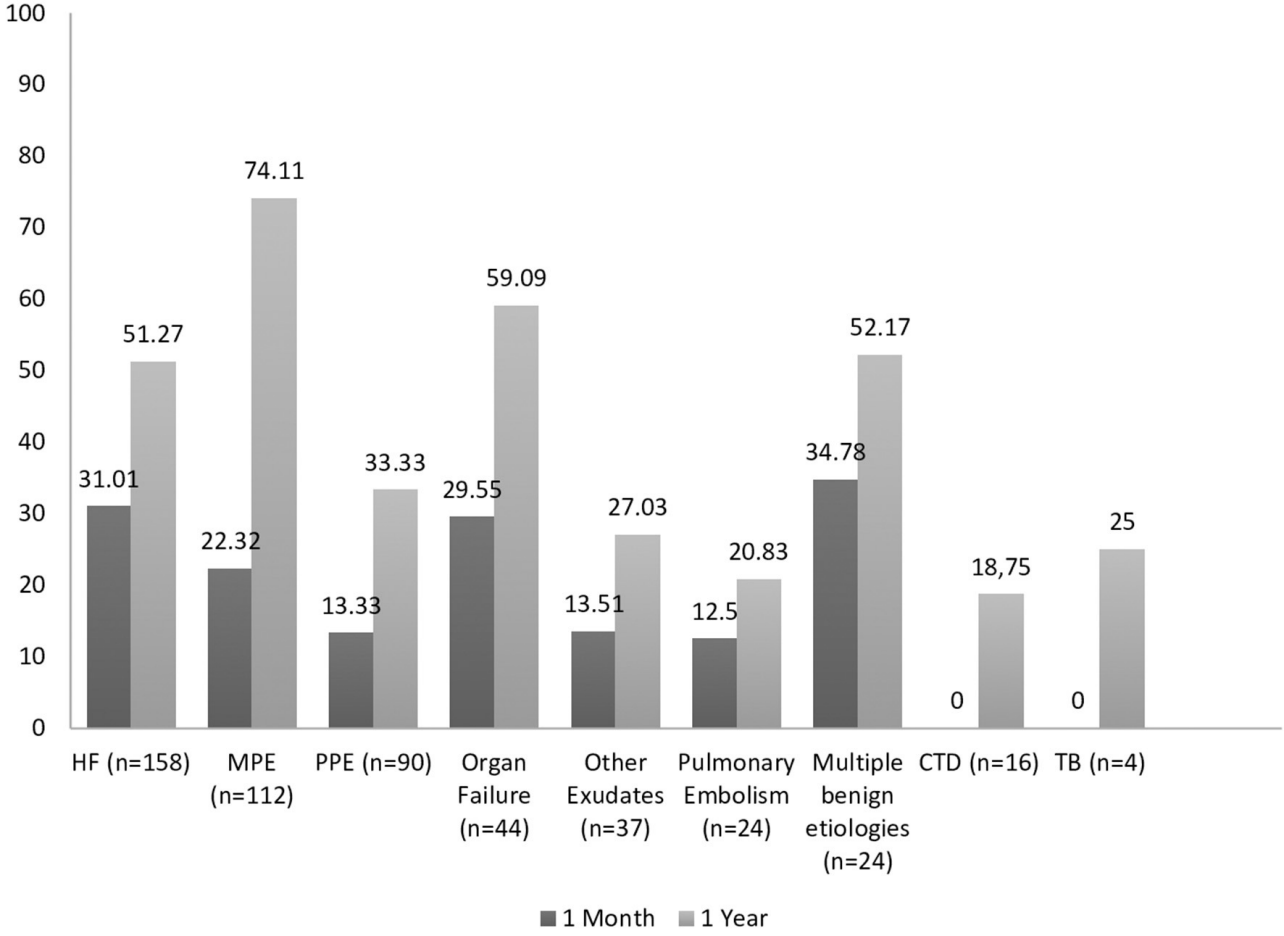
Percent mortality based on the diagnosis of the PE. Short-term mortality was higher for pleural effusions secondary to organ failure, while patients with MPEs experienced the worst prognosis at 1 year.

Mortality rates are illustrated in Figures 1, 2. Overall mortality across all groups was 22.6% (*n* = 115) at 1 month and 49.4% (*n* = 251) at 1 year. Patients with large effusions exhibited higher mortality than patients with small effusions at 30 days (30 vs. 20.9%, *p* = 0.095) and significantly higher at 1 year (66.6 vs. 43.3%, *p* < 0.01). Regarding distribution, patients with bilateral effusion exhibited significantly higher mortality than patients with unilateral effusions at 1 month (32 vs. 13.3%, *p* = 0.005) and higher at 1 year (53.3 vs. 45.5%, *p* = 0.78). Regarding diagnosis, short-term mortality was higher (30–35%) for pleural effusions secondary to organ failure (heart, liver, renal) and multiple benign etiologies, while MPE and other exudates (pleural infection, pulmonary embolism) followed with 22 and 13%, respectively. Patients with MPEs and organ failure experienced the worst prognosis at 1 year (mortality 74 and 51–59%, respectively) while pleural infection followed with 33.3%.

In Tables 3–5, the univariate and multivariate predictors of mortality are displayed. On univariate analysis, significant variables associated with mortality in 30 days were age, CCI, APACHE score, SOFA score, and bilateral distribution. Of note, thoracentesis and CTPA showed a strong negative association with mortality (Table 3). On multivariate analysis, only age, CCI, APACHE score, SOFA score, and bilateral distribution were associated with mortality (Table 5). Regarding long-term mortality, on univariate analysis age, CCI, APACHE score, SOFA score, large size, and malignant etiology predicted mortality, while CTPA showed a protective effect (Table 4). On multivariate analysis that followed, the only significant predictors were CCI, APACHE score, SOFA score, and malignant etiology (Table 5).

**Table 3:** Univariate predictors of mortality at 1 month.

**Sofa score**	**OR**	**95% CI**	***P*-value**
1–2 vs. 0–1	7.636	2.962–19.681	<0.001
2–3 vs. 0–1	22.782	9.056–57.314	<0.001
3–4 vs. 0–1	28.597	10.925–74.855	<0.001
4–5 vs. 0–1	27.908	9.882–78.814	<0.001
>5 vs. 0–1	36.117	11.095–117.573	<0.001
**APACHE II**			
10–14 vs. 0–4	19.563	2.615–146.352	0.004
15–19 vs. 0–4	76.5	10.297–568.342	<0.001
20–24 vs. 0–4	130.768	16.151–1058.793	<0.001
25–29 vs. 0–4	425	23.04–7839.536	<0.001
**Charlson comorbidity index**	1.53	1.319–1.776	<0.001
**Bilateral vs. Unilateral**	3.061	1.957–4.788	<0.001
**Age**	1.048	1.028–1.068	<0.001
**Large vs. Small**	1.627	0.958–2.761	0.072
**CTPA vs. Thorax & abdominal**	0.271	0.103–0.711	0.008
**Thoracentesis**	0.539	0.343–0.846	0.01

*Significant variables associated with mortality were age, CCI, APACHE score, SOFA score, and bilateral distribution. OR, Odds Ratio; CIs, confidence intervals*.

**Table 4:** Univariate predictors of mortality at 1 year.

**Sofa score**	**OR**	**95% CIs**	***p*-value**
1–2 vs. 0–1	2.464	1.506–4.031	<0.001
2–3 vs. 0–1	6.783	3.846–11.965	<0.001
3–4 vs. 0–1	10.577	5.123–21.46	<0.001
4–5 vs. 0–1	8.584	3.807–19.355	<0.001
>5 vs. 0–1	5.519	2.115–14.401	<0.001
**APACHE II**			
5–9 vs. 0–4	1.981	1.047–3.745	0.036
10–14 vs. 0–4	2.829	1.553–5.155	0.001
15–19 vs. 0–4	11.363	5.827–22.156	<0.001
20–24 vs. 0–4	25.566	7.991–81.79	<0.001
**Charlson index**	1.59	1.41–1.794	<0.001
**Large vs. Small**	2.617	1.599–4.281	<0.001
**Age**	1.05	1.035–1.066	<0.001
**HF vs. MPE**	0.368	0.217–0.622	<0.001
**PPE vs. MPE**	0.175	0.095–0.321	<0.001
**Embolism vs. MPE**	0.092	0.031–0.269	<0.001
**CTD vs. MPE**	0.081	0.021–0.303	<0.001
**Exudates vs. MPE**	0.129	0.056–0.3	<0.001
**CTPA vs. other CTs**	0.373	0.187–0.747	0.005

*Significant variables associated with mortality were age, CCI, APACHE score, SOFA score, large size, and malignant etiology. OR, Odds Ratio; Cis, confidence intervals; HF, heart failure; MPE, malignant pleural effusion; PPE, parapneumonic pleural effusion; CTD, connective tissue disease; CTPA, CT pulmonary angiogram*.

**Table 5:** Multivariate predictors of mortality.

**1 month**	**OR**	**95% CIs**	***p*-value**
Age	1.05	1.035–1.066	<0.001
Charlson index	1.53	1.319–1.776	<0.001
Apache 15–19 vs. 0–4	2.912	1.604–5.286	<0.001
Apache 20–24 vs. 0–4	4.277	1.686–10.847	0.002
Apache 25–29 vs. 0–4	17.074	1.741–167.42	0.015
Sofa 1–2 vs. 0–1	5.129	1.942–13.545	0.001
Sofa 2–3 vs. 0–1	9.824	3.589–26.89	<0.001
Sofa 3–4 vs. 0–1	9.726	3.3–28.666	<0.001
Sofa 4–5 vs. 0–1	8.419	2.604–27.217	<0.001
Sofa>5 vs. 0–1	9.883	2.582–37.832	0.001
Bilateral	2.07	1.235–3.471	0.006
**1 year**	**OR**	**95% CIs**	***p*** **-value**
Charlson index	1.303	1.059–1.604	0.012
Apache 15–19 vs. 0–4	2.96	1.617–5.419	<0.001
Apache 20–24 vs. 0–4	7.675	2.426–24.279	0.001
Sofa 2–3 vs. 0–1	2.37	1.264–4.444	0.007
Sofa 3–4 vs. 0–1	3.157	1.406–7.09	0.005
Other exudate vs. MPE	0.077	0.027–0.219	<0.001
HF vs. MPE	0.091	0.039–0.212	<0.001
Organ failure vs. MPE	0.093	0.032–0.268	<0.001
Pulmonary embolism vs. MPE	0.094	0.025–0.35	<0.001
Multiple benign vs. MPE	0.119	0.035–0.407	0.001
PPE vs. MPE	0.182	0.088–0.378	<0.001
Bilateral	1.868	0.989–3.529	0.054
Age	1.026	0.999–1.054	0.063
Large vs. small	1.771	0.955–3.287	0.07

*Age, CCI, APACHE score, SOFA score, and bilateral distribution were associated with mortality in 1 month, while CCI, APACHE score, SOFA score, and malignant etiology were associated with mortality in 1 year. OR, Odds Ratio; CIs, confidence intervals; HF, heart failure; MPE, malignant pleural effusion; PPE, parapneumonic pleural effusion*.

We also analyzed subjects who underwent thoracentesis (Table 6). Exudates excluding MPEs exhibited a survival benefit at both 1 month and 1-year observations. Due to the smaller *n* sample, fluid characteristics were not included in the multivariate analysis.

**Table 6:** Transudates vs. exudates (excluding MPEs) on mortality.

**1 Month**	**OR**	**95% Cis**	***p*-value**
Exudate vs. Transudate	0.209	0.075–0.585	0.003
**1 Year**			
Exudate vs. Transudate	0.219	0.098–0.488	<0.001

*Exudates excluding MPEs exhibited a survival benefit at both 1-month and 1-year observations. OR, Odds Ratio; CIs, confidence intervals*.

A separate analysis of solely MPEs is depicted in Table 7. Cox proportional hazards regression analysis identified high APACHE score and bilateral distribution as the factors associated with worse survival among MPEs.

**Table 7:** Prognostic characteristics of survival in subjects with Malignant pleural effusions.

**Variables**		**Hazard ratio**	**95% confidence intervals**	***p***
Age	72 (67–81)	1.015	0.9885–1.043	ns
Female sex	36 (32)	1.092	0.5813–1.964	ns
Smoking	84 (76)	0.97	0.4792–1.941	ns
Unilateral/Bilateral PE	92 (83)/19 (17)	3.49	1.700–6.969	0.0005
Small/moderate/large PE	25 (22)/44 (40)/42 (38)	0.76	0.4613–1.253	ns
APACHE II score	10 (5–15)	1.06	1.005–1.125	0.035
SOFA score	2 (1–3)	0.86	0.6845–1.056	ns
CCI	4 (4–5)	1.12	0.9076–1.385	ns
In-hospital days	8 (5–15)			
Survival days from diagnosis	100 (39–339)			

*Continuous variables are presented as median (interquartile range) and categorical outcomes as absolute n (% frequency). Survival analysis is performed using Cox proportional hazards regression (significant p < 0.05). PE, pleural effusion; APACHE II, acute physiology and chronic health evaluation; SOFA, sequential organ failure assessment; CCI, Charlson comorbidity index*.

**Figure 3:**
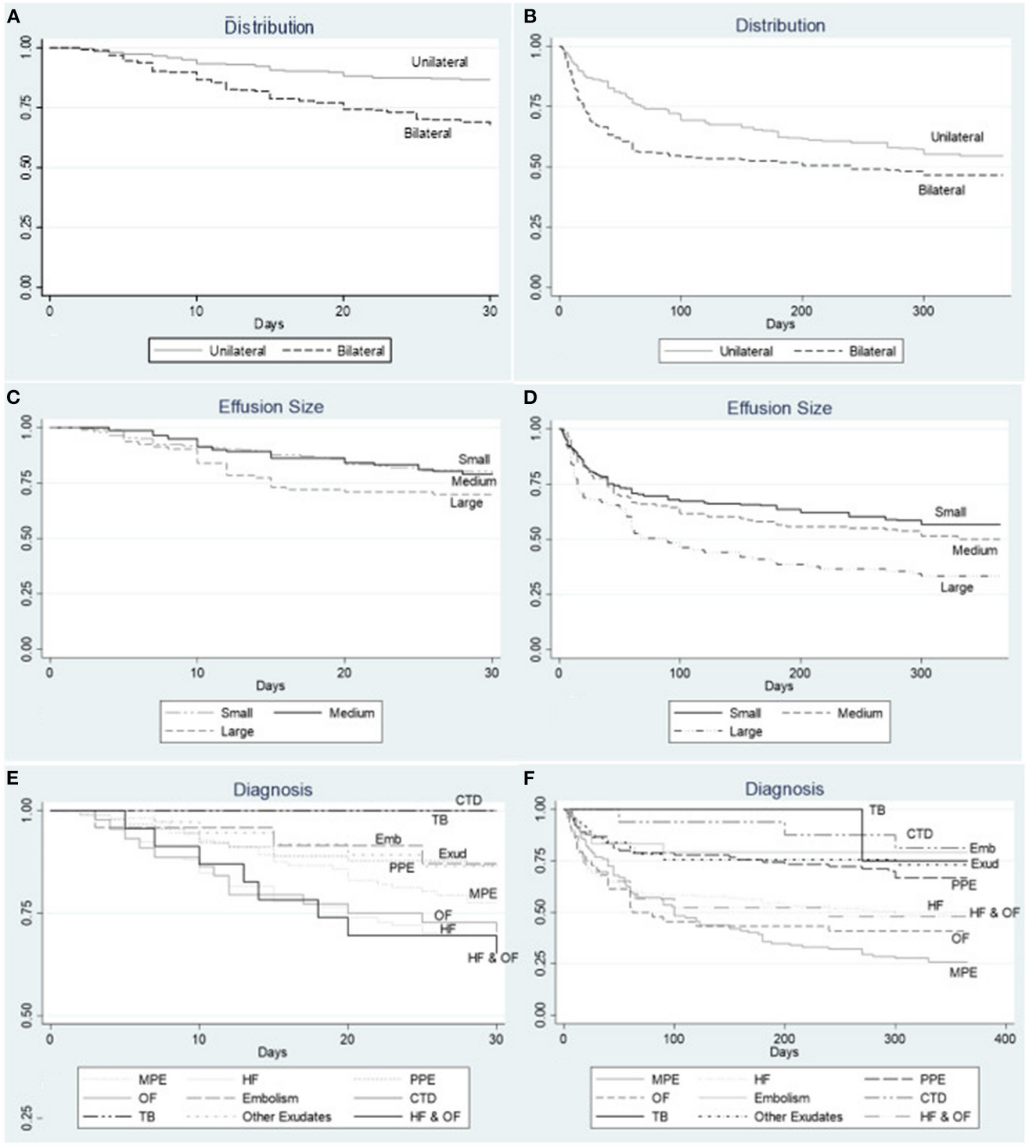
Kaplan Meier survival curves at 1 month and 1 year by **(A,B)** distribution of PE. In both time periods, the presence of bilateral pleural effusion was associated with lower survival probability. **(C,D)** size of PE. In both time periods, the presence of large pleural effusion was associated with lower survival probability. **(E,F)** diagnosis of PE. Short-term survival is lower for patients with pleural effusions secondary to organ failure (heart, liver, renal) and multiple benign etiologies, while long-term survival is worse for patients with MPE. HF, heart failure; MPE, malignant pleural effusion; PPE, parapneumonic pleural effusion; CTD, connective tissue disease; Emb, pulmonary embolism; OF, organ failure; Exud, other exudate; TB, tuberculosis.

Figure 3 contrasts the Kaplan-Meier survival curves by distribution, size, and diagnosis of PE:

(a,b) Distribution of PE. In both time periods, the presence of bilateral pleural effusion was associated with lower survival probability.
(c,d) Size of PE. In both time periods, the presence of large pleural effusion was associated with lower survival probability.
(e,f) Diagnosis of PE. Short-term survival is lower for patients with pleural effusions secondary to organ failure (heart, liver, renal) and multiple benign etiologies, while long-term survival is worse for patients with MPE.

## Discussion

We performed a multicenter prospective observational study and demonstrated that PEs carry significant morbidity and mortality. Among all clinical parameters studied, short-term mortality was associated in our study with increased age, bilateral effusions, APACHE II and SOFA scores, and a high Charlson comorbidity index. Long-term mortality was found associated with a high Charlson comorbidity index, APACHE II and SOFA scores, and the presence of malignant pleural effusion. Overall mortality in our study was 22, 6% at 1 month and 49, 4% at 1 year, similar to previous findings of Debiasi and colleagues (21% at 30 days and 51% at 1 year). Kookolis and colleagues in a retrospective study reported overall mortality of 15% at 30 days and 32% in 1 year (9, 10). These findings taken together illustrate a significant burden of pleural effusions in patients needing hospitalization in pulmonary or in other departments.

APACHE II score ranging from 10 to 14 is found to be associated with 7–15% in-hospital mortality (13), Sofa score above 2 is related to an increased risk of in-hospital mortality (14), and Charlson comorbidity index above 5 is associated with 80% 10 year-mortality (15). In our study worse APACHE II and SOFA scores were significant predictors of both short-term and long-term mortality. This was also demonstrated by Kookoolis et al. (9). On the other hand, a novel finding of our study is the association of the Charlson comorbidity index with mortality. Thus, our findings suggest that the occurrence of pleural effusion in an aged individual with already multiple comorbidities may lead to acute decompensation as demonstrated by clinical severity scores. Therefore, these patients upon admission should be monitored closely.

Congestive heart failure (HF) is the most common cause of PE (16) however the prognostic role of HF-related effusions is not well-established. In a prospective study of 100 patients, PE didn't predict outcome or mortality during a 6-month follow-up (17). Ercan and colleagues reported favorable survival (81% at 1 year, *n* = 151) when effusions were incidentally observed in transthoracic echocardiogram (18). However, recent prospective studies report high mortality at 1 year (near 50%), suggesting that HF-related PEs, especially large refractory cases requiring aspiration, have a poor prognosis (10, 11).

Regarding other benign etiologies, mortality rates are also high. Walker and colleagues reported that 25% of patients with liver failure die within 1 year (11). In a population-based study of 3.487 cirrhotic patients with PE requiring drainage, 30-day and 1-year mortality were 20.1 and 59.1%, respectively (19). Mortality in PE associated with renal etiology is not well-studied, yet a study of a small cohort of 14 patients with renal failure showed 14 and 57% 30-days and 1-year mortality, respectively (10). We report here significant high mortality rates for all patients with organ failure (20–30% in 1 month and 50–60% in a year).

Malignant pleural effusion (MPE) affects almost 15% of patients with underlying malignancy and is associated with a poor life expectancy (20). Like other studies, we demonstrated that MPE is associated with high mortality rates; 22% at 30 days and 74% at 1 year. Regarding long-term outcomes, patients with MPE had the worse prognosis of all underlying etiologies. Among MPEs we found bilateral distribution and high APACHE score, indicating acute but also chronic health decompensation, associated with worse outcomes. Given this poor outcome, prognostic tools are crucial to personalize treatment and avoid unnecessary interventions (6, 21).

It has been documented that PEs are poor prognostic signs in patients with pulmonary infection, especially when they are large, bilateral, or associated with empyema (4, 22, 23). Mortality rates range from 1% in simple uncomplicated parapneumonic pleural effusions to 30% in empyema or even 50% in ICU patients (8, 24–26). Our study shows a significant risk of death in hospitalized patients with pleural infection (13.3% at 30 days), however exudative effusions had a favorable prognosis as opposed to transudative effusions (Table 6).

It has been established that the presence of bilateral PEs in patients with community-acquired pneumonia is an independent predictor of 30-day mortality with a relative risk of 2.8 (22). However, Debiasi and colleagues first reported the association between bilateral PEs of any etiology and mortality. They reported 1-month mortality rates of 17% for unilateral vs. 36% for bilateral PEs, and 1-year mortality rates 47 and 69%, respectively (10). Similarly, Walker and colleagues reported 1-year mortality rates of 20 and 57% for unilateral vs. bilateral effusions (11). In accordance with these findings, we reported 1-month mortality rates of 13.3% for unilateral vs. 32% for bilateral effusions. At 1 year our rates increase to 45.5 and 53.4%, respectively. Bilateral PEs in our study reflect the increased mortality rates observed in heart, liver, kidney, or multi-organ failure patients. Therefore, the presence of bilateral PE regardless of etiology predicts significant mortality.

We also report a possible negative association between thoracentesis and mortality at 30 days. Kookoolis and colleagues first documented a protective role of thoracentesis in a retrospective cohort. Existing guidelines don't recommend thoracentesis in patients within a clinical context highly suspicious of transudative PE (27). Our finding might be due to underlying exudative etiologies, necessitating thoracentesis more commonly than transudates, since in our study exudates as we already mentioned had a better prognosis than transudates. We may not make a conclusive comment regarding the significance of thoracentesis in the present study, since not all effusions were aspirated. Further, undergoing thoracentesis may be a confounding signal reflecting the patient's clinical status allowing a procedure or not.

The same applies to CTPA that also showed a protective role since CTPA is usually performed in unilateral PEs in patients with lower clinical severity scores and underlying exudative etiologies (e.g., pulmonary embolism). Inhomogeneous CT requirement for inclusion in this study might introduce recruitment and confirmation bias, with mode of CT selected dependent on clinical and laboratory subjects' condition. Therefore, the clinical utility of each CT mode cannot be commented in our study. We believe however that this method allowed us to include more compromised patients and to better quantify the pleural effusion.

To our knowledge, this study is the largest prospective study on mortality in hospitalized patients with PE regardless of etiology and thoracentesis or not. Charlson comorbidity index, clinical severity scores, bilateral distribution, and malignancy reflect on mortality of PEs. As to the limitations of our study, our cohort represents hospitalized patients thus our results cannot be generalized to an outpatient setting. The limited number of subjects that underwent thoracentesis did not allow effusion discrimination by Light's criteria to be included in the multivariate analysis.

## Conclusion

Pleural effusion is a marker of advanced disease. In our study, 20% of hospitalized patients died within 30 days and almost 50% within a year. Mortality tops within the first month in patients with pleural effusions related to organ failure, while patients with malignant pleural effusions have the worst long-term outcome. Independent predictors of mortality, apart from the Charlson comorbidity index, APACHE score, and SOFA score, are age and bilateral distribution in the short term and malignancy in the long term. Transudative effusions are possibly associated with worse outcomes.

## Data Availability Statement

The original contributions presented in the study are included in the article/supplementary material, further inquiries can be directed to the corresponding author/s.

## Ethics Statement

The studies involving human participants were reviewed and approved by Institutional Ethics Committee of University Hospital of Larissa (Ethical Committee Approval Number: 15/12-02-2019). The patients/participants provided their written informed consent to participate in this study.

## Author Contributions

EM, IP, and KG were responsible for the conceptualization and methodology of the study. EM, GP, AA, KP, CV, and EC were responsible for the collection of the samples. EM and IP evaluated the results and data and were responsible for the writing. KG was the supervisor of the study. All authors have read and agreed to the published version of the manuscript.

## Conflict of Interest

The authors declare that the research was conducted in the absence of any commercial or financial relationships that could be construed as a potential conflict of interest.

## Publisher's Note

All claims expressed in this article are solely those of the authors and do not necessarily represent those of their affiliated organizations, or those of the publisher, the editors and the reviewers. Any product that may be evaluated in this article, or claim that may be made by its manufacturer, is not guaranteed or endorsed by the publisher.
